# Influence of celecoxib on the vasodilating properties of human mesenteric arteries constricted with endothelin-1

**DOI:** 10.3892/br.2014.233

**Published:** 2014-01-29

**Authors:** GRZEGORZ GRZEŚK, KATARZYNA SZADUJKIS-SZADURSKA, GRZEGORZ MATUSIAK, BARTOSZ MALINOWSKI, MARTA GAJDUS, MICHAŁ WICIŃSKI, LESZEK SZADUJKIS-SZADURSKI

**Affiliations:** Department of Pharmacology and Therapeutics, Collegium Medicum, Nicolaus Copernicus University, Bydgoszcz 85-094, Poland

**Keywords:** endothelin-1, celecoxib, phosphodiesterase inhibitors, constriction, endothelium

## Abstract

The mitogenic and vasoconstrictive properties of the vascular system are attributed to endothelin-1 (ET-1). ET-1 serum concentration increases in a number of pathological conditions, particularly in those associated with blood vessel constriction. ET-1 is also associated with the underlying pathomechanisms of primary pulmonary hypertension, arterial hypertension and eclampsia. The aim of this study was to compare the vasodilating properties of selected phosphodiesterase (PDE) inhibitors and celecoxib in human mesenteric arteries constricted with ET-1, and investigate the role of the endothelium in relaxation. Perfused human mesenteric arteries were collected and stored under the same conditions as organs for transplantation. The mesenteric arteries (with and without the endothelium) were constricted by the addition of ET-1 and treated with one of the following: sildenafil (PDE5 inhibitor), zaprinast (PDE5 and 6 inhibitor), rolipram (PDE4 inhibitor) and celecoxib [cyclooxygenase-2 (COX-2) inhibitor]. Based on the observed changes of the perfusion pressure, concentration response curves (CRCs) were prepared for the respective inhibitors and the EC_50_ (concentration causing an effect equal to half of the maximum effect), pD_2_ (negative common logarithm of EC_50_) and relative potency (RP) were calculated. The results suggested that all the inhibitors triggered a concentration-dependent decrease in the perfusion pressure in isolated human superior mesenteric arteries with endothelium constricted by the addition of ET-1. In the arteries without endothelium, CRCs for celecoxib and rolipram were shifted to the right without a significant decrease in the maximum dilating effect. Moreover, CRCs for sildenafil and zaprinast were shifted to the right with a simultaneous significant decrease in the maximum dilating effect and with an increased inclination angle in reference to the concentration axis. In the presence of the endothelium, all of the evaluated PDE inhibitors, as well as celecoxib, reduced the reactivity of the mesenteric arteries caused by ET-1. Sildenafil indicated the lowest efficacy in the presence of the endothelium, but showed a higher potency compared to that of the other compounds. Removing the endothelium significantly reduced the vasodilating efficacy of PDE5 and 6 inhibitors and a statistically significant influence on the vasodilating efficacy of PDE4 inhibitor and celecoxib was observed. The high vasorelaxing efficacy of celecoxib at the background of the PDE inhibitors was observed, not only in the presence, but also in the absence of the endothelium and may be evidence for the relaxation induced by this COX-2 inhibitor in the cAMP- and cGMP-dependent pathways.

## Introduction

Due to numerous enzymes and receptors, the endothelium is an active platform for the interaction between blood (including signaling substances carried in the blood) and the vessel wall, playing a fundamental role in regulating vasomotor activity, hemostasis and angiogenesis, as well as in controlling inflammatory and immune processes. Key characteristics allowing the endothelium to perform these functions include speed in the stimulation of its cells and their response to stimuli.

Efficient vasoconstriction and vasodilatation, as well as the resulting influence on the arterial pressure and blood supply to tissues depend on a number of substances secreted by the endothelium in response to stimulation by shear stress, hypoxia and acetylcholine, bradykinin or serotonin action. Vasoactive substances of endothelial origin include the group with vasoconstrictive properties [such as endothelin, angiotensin II, platelet-activating factor, thromboxane A_2_ (TXA_2_), leukotriene A_4_ and B_4_, ATP and ADP] and the group, which stimulates dilation of the vessels [such as nitric oxide (NO), prostacyclin (PGI_2_), vasodilator-stimulated phosphoprotein, endothelium-derived hyperpolarizing factor and adenosine as a product of the membranous ectonucleases degrading ATP and ADP] (1). Due to secretion of NO, the endothelium is also responsible for paracrine antioxidative action (limiting oxidation of low-density lipoproteins on the subendothelial space) and antiproliferative action (inhibiting mitogenesis of the smooth muscle cells). PGI_2_ diffusing into the vessel lumen determines the antiaggregant action of the endothelium on the platelets.

Mitogenic and vasoconstrictive properties are attributed to endothelin-1 (ET-1) in the vascular system. The serum concentrations of ET-1 increase in a number of pathological conditions, particularly in those associated with blood vessel constriction. ET-1 is also associated with the underlying pathomechanisms of primary pulmonary hypertension, arterial hypertension and eclampsia.

Cyclooxygenase (prostaglandin G/H synthase) is an enzyme transforming arachidonic acid to cyclic peroxides (prostaglandin G_2_ and H_2_), which are unstable reaction intermediates and precursors of TXA_2_, PGI_2_ and other prostaglandins (PGE_2_, PGF_2_ and PGD_2_). Two forms of cyclooxygenase were isolated, cyclooxygenase-1 (COX-1) and COX-2. COX-1 is mainly a constitutive isoform, which is present in the majority of cells and tissues; however, certain cytokines, growth factors, mitogens, ischemia and several other chemical and physical damaging factors induce COX-2 synthesis. COX-1 may maintain functions, such as cytoprotection of the gastric epithelium and COX-2 is the main source of prostanoids during inflammation or the development of neoplasms. However, in certain tissues, COX-2 is also a constitutive enzyme (in the brain and kidney tissues) and is produced in the endothelium as a result of laminar friction force constituting a crucial link in the process of vascular tone adaptation. Therefore, the assumption that COX-2 is the enzyme independently responsible for inflammatory processes currently appears to be incorrect. Previous studies on COX-2 knockout mice reported that COX-1 was able to produce a sufficient amount of prostaglandins to induce a full inflammatory response (2). In the heterological system of expression, COX-1 preferentially binds with TXA_2_ synthase and PGF_2_ synthase, whereas COX-2 preferentially binds with PGI_2_ synthase (3).

All eicosanoid receptors belong to a group of receptors bound to G protein known as G protein-coupled receptors (GPCRs). Activation of GPCRs results in the modulation of adenylyl cyclase and phospholipase C activity due to the well-documented role of cyclooxygenases in inflammatory processes, amplification processes of pain sensations and in body temperature regulation. These enzymes are an attractive target for pharmacological symptomatic treatment. Non-steroidal anti-inflammatory drugs (NSAIDs), acetylsalicylic acid and acetaminophen interact with COX-1 and COX-2.

The hypothesis that anti-inflammatory effects may be separated from an ulcerogenic effect encouraged investigators to assess agents with a higher selectivity to COX-2 than to COX-1. A group of compounds referred to as coxibs, which currently include celecoxib, rofecoxib, etoricoxib, valdecoxib, lumiracoxib and several others were identified. Further studies reported that their selectivity with regards to COX-2 is similar to the selectivity of previously identified NSAIDs, such as meloxicam, nimesulide and diclofenac. *In vivo*, selective COX-2 inhibitors reduce the production of PGI_2_ by endothelial cells without simultaneous inhibition of the platelet thromboxane. Thus, selective COX-2 inhibitors may increase the risk of a thrombus (4–7). Furthermore, animal studies conducted on mice and extensive epidemiological data suggest that the probability of the occurrence of hypertension caused by NSAIDs reflects a degree of COX-2 inhibition and selectivity of this process. Constitutive activity of COX-2 was reported in the vascular endothelial cells in patients with sclerosis or diabetes mellitus (2) and increased concerns as regards the safety of using this group of agents in humans (8).

Celecoxib, which has an affinity to COX-2 that is ~375-fold higher compared to its affinity to COX-1, was registered for the symptomatic treatment of inflammation and pain in patients with osteoarthritis, rheumatoid arthritis and ankylosing spondylitis. It is also used to reduce the number of adenomatous polyps in patients with familial adenomatous polyposis, as a therapy for supporting surgical treatment and for further endoscopic control (9).

The number of available studies on the role of celecoxib in modulating receptors of intracellular signaling pathways, which eliminate endothelin-dependant vascular constriction, is currently limited. The present study compared celecoxib and substances with known vasodilating properties (considering the dependence of these properties on the presence of the endothelium) in a series of experiments on mesenteric arteries using ET-1 to induce vascular constriction.

Investigating the methods for antagonizing constrictive properties of endothelin by modifying intracellular signaling pathways of the vascular smooth muscles and studying their dependence on paracrine activity of the endothelium may contribute to more effective treatment of the aforementioned diseases and aid in the search for novel methods to prevent their complications.

This study aimed to compare the vasodilating properties of selected phosphodiesterase (PDE) inhibitors and celecoxib in human mesenteric arteries constricted with ET-1, as well as investigate the role of the endothelium in relaxation.

## Materials and methods

### Materials and reagents

Human mesenteric arteries were obtained from subjects whose organs were collected for transplantation and stored under the same conditions. Human superior mesenteric arteries were collected in compliance with binding legal regulations. The Bioethics Committee at the Collegium Medicum, Nicolaus Copernicus University (Bydgoszcz, Poland) provided consent for conducting this experiment (KB/344/2005 and KB/252/2008). The patient or the patients’ family gave informed consent. All the reagents used in this study were purchased from Sigma-Aldrich (Poznan, Poland).

### Treatment of perfused human mesenteric arteries with endothelium

In the first series of experiments, the mesenteric arteries with a maintained endothelium, constricted by the addition of ET-1 (6.5×10^−9^ mol/L) were treated with increased concentrations of the following: sildenafil (PDE5 inhibitor; 0.1×10^−9^ to 1×10^−6^ M), zaprinast (PDE5 and 6 inhibitor; 3×10^−8^ to 3×10^−4^ M), rolipram (PDE4 inhibitor; 3×10^−7^ to 3×10^−3^ M) and celecoxib (COX-2 inhibitor; 3×10^−9^ to 3×10^−5^ M). Based on the observed changes in perfusion pressure, concentration response curves (CRCs) were prepared for the respective inhibitors and the following were calculated: EC_50_ (concentration causing an effect equal to half of the maximum effect), pD_2_ (negative common logarithm of EC_50_ value) and relative potency (RP), i.e., quotient of EC_50_ control value and EC_50_ value analyzed in the experimental systems. This series of experiments facilitated the comparison of the efficacy of selected PDE inhibitors and celecoxib in the dilation of mesenteric arteries.

### Treatment of perfused human mesenteric arteries without endothelium

The next series of experiments were conducted in a manner similar to the aforementioned one; however, prior to the experiment, the endothelium was removed from the vessels using compressed air in accordance with the methodology developed by Koller *et al* (10). Precision of endothelium removal was verified using a perfusate containing acetylcholine chloride in a concentration of 1×10^−5^ M. The occurrence of constriction of the vessel was recognized as confirmation that the endothelium was absent. This series of experiments facilitated the comparative evaluation of the efficacy of selected PDE inhibitors and celecoxib in the dilation of mesenteric arteries and the influence of the endothelium.

### Statistical analysis

Statistical analysis was performed by calculating the mean values and standard deviations. The results are presented as the means of serial measurements with consideration of the standard error of the mean. P<0.05 was considered to indicate a statistically significant difference. Values of 0.05≤P<0.1 expressed a trend towards statistical significance, but values of P≤0.1 were not significant.

## Results

### PDE inhibitors and celecoxib decreased the perfusion pressure in human mesenteric arteries with endothelium

**Figure 1:**
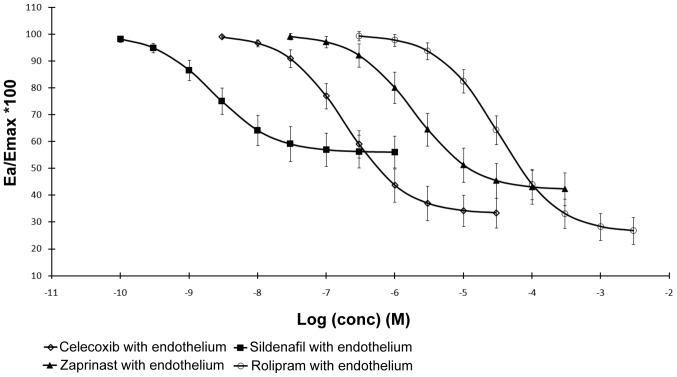
CRCs for celecoxib, zaprinast, sildenaphil and rolipram. The study was performed on human mesenteric arteries (with endothelium) contracted by ET-1. All the inhibitors triggered a concentration-dependent decrease in perfusion pressure in the mesenteric arteries. Points marked on the CRC present the mean relaxation effect in % and SE (n=12 arteries per group). Graphs were approximated to sigmoidal curve. CRC, concentration response curves; ET-1, endothelin-1; SE, standard error; Emax, maximal response produced by the drug.

The series of experiments conducted on perfused human mesenteric arteries with a maintained endothelium revealed that all the PDE inhibitors and celecoxib triggered a concentration-dependent decrease in perfusion pressure in isolated arteries constricted by ET-1 (Fig. 1). The PDE inhibitors and COX-2 inhibitor indicated characteristics of non-competitive (functional) antagonists and did not completely eliminate vascular constriction caused by ET-1 (Fig. 3). The basic pharmacometric parameters of human mesenteric arteries (with and without endothelium) treated with PDE inhibitors and celecoxib and constricted by ET-1 are summarized in Table I.

### Analysis of PDE inhibitors and celecoxib in human mesenteric arteries without endothelium

**Figure 2:**
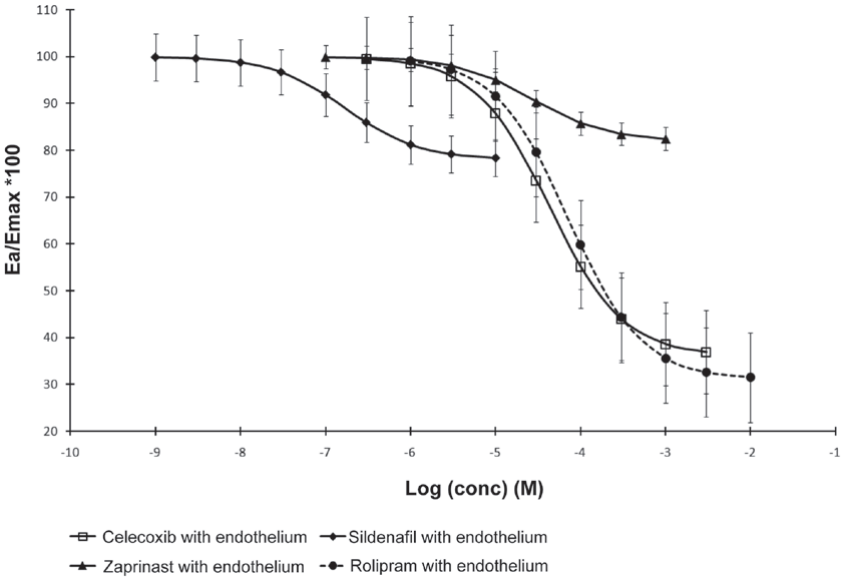
CRCs for celecoxib, zaprinast, sildenafil and rolipram. The study was performed on human mesenteric arteries (without endothelium) contracted by ET-1. Points marked on the CRC present the mean relaxation effect in % and SE (n=12 arteries per group). Graphs were approximated to the sigmoidal curve. CRCs, concentration response curves; ET-1, endothelium; SE, standard error.

**Figure 3:**
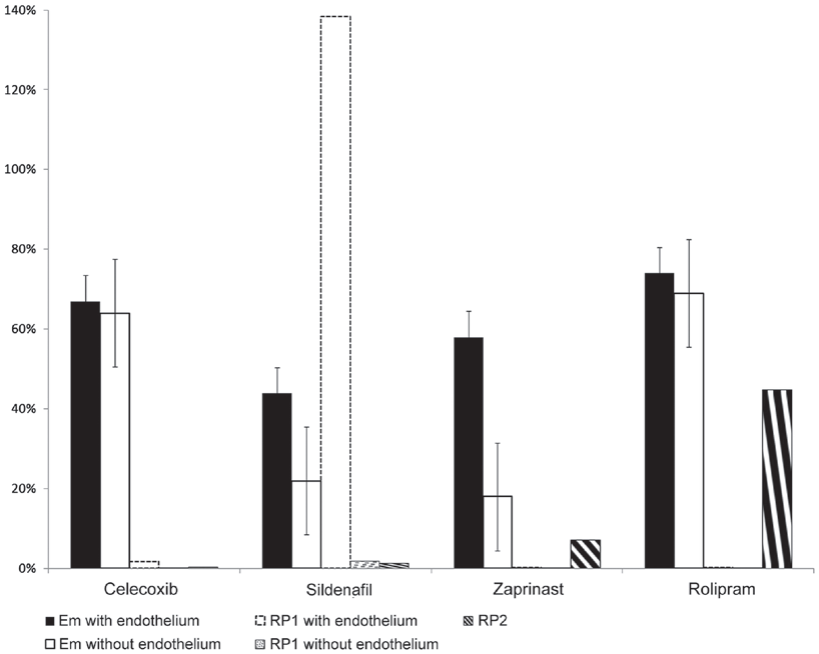
Em and RP of celecoxib, sildenafil, rolipram and zaprinast for human mesenteric arteries, with and without the endothelium constricted by ET-1. Results are based on the data from Table I. Em, maximum effect; RP, relative potency.

The series of experiments conducted on the arteries with removed endothelium showed that CRCs for celecoxib and rolipram were shifted to the right without a statistically significant decrease in the maximum dilating effect of celecoxib and the PDE4 inhibitor (Fig. 2). Furthermore, CRCs for sildenafil and zaprinast were shifted to the right, but with a simultaneous statistically significant decrease in the maximum dilating effect of PDE5 and PDE5/6 inhibitors and with an increased inclination angle in reference to the concentration axis (Fig. 2). The PDE inhibitors and COX-2 inhibitor demonstrated characteristics of non-competitive (functional) antagonists in vessels without an endothelium and did not completely eliminate vascular constriction caused by ET-1.

### Comparative analysis of CRCs for PDE inhibitors and celecoxib in human mesenteric arteries with and without endothelium

Comparative analysis of the CRCs for the PDE inhibitors and the COX-2 inhibitor in mesenteric arteries with and without the endothelium suggested that removal of the endothelium corresponds to a model of competitive inhibition in reference to the vasodilating effects of celecoxib and rolipram and non-competitive inhibition in reference to the vasodilating effects of zaprinast and sildenafil.

## Discussion

A recognized method for limiting the use of vasodilative factors is inhibiting the decomposition of cyclic monoribonucleotides, cAMP and cGMP. Cyclic nucleotides, cAMP and cGMP, are synthesized intracellularly by adenylyl and guanylyl cyclases (11–13) in the presence of Mg^2+^ ions. They activate protein kinases, PKA and PKG, regulate the intracellular Ca^2+^ concentration and influence the function of ion channels (14–16). An increased level of cyclic nucleotides results in a decreased concentration of cytoplasmic Ca^2+^, decreased sensitivity of the smooth muscles to calcium and consequently leads to dilation (17,18).

The concentration of cGMP in vascular smooth muscle cells is associated with the condition of the endothelium. A stimulated endothelium produces NO, which diffuses into the muscular layer and stimulates cGMP production by covalently binding to a heme group of soluble guanylyl cyclases (sGC) (19,20). It has been reported that aquaporin-1 transports NO through the cell membranes (21) and the synthesis of aquaporins is inhibited by ET-1 (22). However, Slupski et al (23) reported that sodium pump stimulation by YC-1, as an additional mechanism of sGC activation independent of cGMP, relaxed human mesenteric arteries, including blockade of calcium ion influx.

The influence of the endothelium on reserves of cAMP is much less explicit. Although locally produced PGE_2_ and PGI_2_ reduce the tone of the blood vessel walls by GPCRs (IP and EP), the endothelium is also recognized as a potential source of PGH_2_, a vasoconstricting prostanoid, which acts by reducing the concentration of cAMP in the smooth muscle cells. PGI_2_, the main metabolite of the arachidonic acid released from the vascular endothelium, is mainly produced in humans with the participation of COX-2 (24). PGH_2_ synthesis is controlled by shear stress and autacoids, which constrict and dilate vessels. A previous study suggested that the role of PGI_2_ in the local regulation of vascular tone was not significant, but studies analyzing the polymorphism of PGI_2_ found an association between the risk of myocardial infarction and severe hypertension (3). Simultaneously, it was reported that PGI_2_ limits pulmonary hypertension induced by hypoxia and general hypertension caused by angiotensin II (25). PGE_2_, produced in the endothelium with the participation of COX-2 and through the EP_4_ receptor, maintains the patent arterial duct until birth, when decreased levels of PGE_2_ result in its closing (26). Discrepant observations in the case of PGE_2_ may result from the multitude of receptors for this prostanoid.

The present study compared celecoxib, a COX-2 inhibitor with unique properties of inhibiting PDE5 and 4, with classical PDE inhibitors occurring in the endothelium (PDE4, 5 and 6). Our results suggested that superior mesenteric arteries with a maintained endothelium and constricted with ET-1 responded to the vasodilative action of all the PDE inhibitors as well as celecoxib. With regards to sildenafil, a reduced perfusion pressure by ~44% of the maximum effect of ET-1 was achieved. Zaprinast and rolipram were found to be more effective in causing dilation. In the mesenteric arteries with a maintained endothelium, celecoxib triggered a concentration-dependent reduction in perfusion pressure reaching 67% of the maximum effects of ET-1 and this result did not statistically differ from those of rolipram and zaprinast. Sildenafil showed the lowest EC_50_ value of 2.29 (±0.04) × 10^−9^ M/L. In addition, low concentrations of PDE inhibitors, celecoxib and other studied substances were not able to reach dilation efficacy, which was equal to the efficacy of sildenafil.

The reduced myorelaxing efficacy of the PDE5 and 6 inhibitors was notable while comparing the results obtained in arteries with endothelium and those without endothelium. Our results suggested that cAMP plays a crucial role in vasoreactivity of the choke vessels, but it is mainly independent from the endothelium. Celecoxib is a poor PDE4 inhibitor and showed its ability to increase cAMP at high concentrations; however, at lower concentrations it efficiently acts through PDE5 inhibition. Thus, vasorelaxant pathways based on cGMP may be referred to as the pathways controlled by the presence and condition of the endothelium, and vasorelaxant pathways based on cAMP may be referred to as the endothelium-independent pathways.

The vascular effects of celecoxib have been approached carefully. Data provided by two large, randomized and double-blinded clinical studies with the use of celecoxib (Celecoxib Long-term Arthritis Safety Study) and rofecoxib [Vioxx^®^ Gastrointestinal Outcomes Research (VIGOR)] in patients with chronic arthritis, despite the fact that fewer adverse events associated with an alimentary tract in the group of patients taking coxibs compared to subjects taking non-selective NSAIDs was observed, detailed analysis of the results confirmed doubts regarding the safety of coxibs in reference to their influence, not only on the mucous membrane of the alimentary tract, but also on the cardiovascular system (8,27–29). VIGOR reported an increased risk in the occurrence of cardiovascular events in the group of patients taking rofecoxib, which led to its recall by the Food and Drug Administration in December, 2004. In addition, previous studies have reported that celecoxib increased the incidence of cardiovascular complications compared to that of the placebo, but did not increase general mortality (30,31). An explanation for the adverse events caused by COX-2 inhibitors was ascribed to the action of these drugs resulting in the decreased synthesis of PGI_2_, which reveals vasodilating and anti-aggregating properties previously known. *In vivo*, decreasing the synthesis of PGI_2_ may have been associated with superior influence of its functional antagonist, thromboxane (7,32).

While the safety of coxibs, which in the light of previous data confirming increased cardiovascular risks may raise doubts, the vasodilating action of celecoxib itself is well-documented. Animal studies on guinea pigs and rats reported that celecoxib dilated coronary arteries in guinea pigs and large arteries in rats by intensifying the effects of NO/cGMP associated with the inhibition of PDE5 (33). In these studies, the vasodilating efficacy of celecoxib was found to be reduced compared to that of sildenafil, but higher compared to that of zaprinast. It was also reported that the vasodilating efficacy of celecoxib was limited by the NO synthase inhibitor (L-NAME) and guanylyl cyclase inhibitor. In direct measurements, celecoxib increased the levels of cGMP in the smooth muscle treated with sodium nitroprusside at a concentration of 5×10^−7^ M/L by ~5-fold. These unexpected actions of celecoxib were explained with PDE5 block, which significantly compensated for the decrease of cAMP associated with the assumed reduction in PGI_2_ synthesis. EC_50_ of celecoxib was also established in relation to human PDE5_A1_ at 1.6×10^−5^ M/L.

Previous studies have reported that the use of celecoxib is associated with a reduced risk in developing or losing control of hypertension, as well as with a reduced risk of acute cardiovascular events, than it is with selective COX-2 inhibitors, in particular, rofecoxib (31,34,35). It was suggested that celecoxib may reduce endothelial dysfunction (16,36,37). Studies using recombinant human PDE have indicated that celecoxib also inhibited the activity of PDE4 (EC_50_=1×10^−5^ M/L), but did not influence PDE1, 2 and 3. It was emphasized that the effects of low intracellular concentrations of PGI_2_-dependent cAMP caused by celecoxib may be compensated by increased cGMP, inhibition of PDE3 by cGMP and by the direct inhibition effects of celecoxib on PDE4 (33). These properties have not been confirmed on other selective COX-2 inhibitors except for valdecoxib, which may inhibit PDE5, but more weakly compared to that of celecoxib (2). It is vital that the vasodilating properties of celecoxib were also observed *in vivo*. An increase in blood flow in the brachial artery was identified in patients with hypertension following treatment with celecoxib in a placebo-controlled trial (38). This supports the hypothesis that celecoxib restores endogenous vasorelaxing sensitivity.

Recently, it was reported that selective COX-2 inhibitors restored, not only the vasodilating response of sclerotically degenerated arteries, but also the vasoconstricting response. Experimental data provided by studies evaluating sclerotically degenerated arteries in rabbits suggested an increased sensitivity to noradrenalin to the level comparable to the sensitivity of normal arteries following the use of indomethacin. Lowered PGI_2_ synthesis was observed under the influence of the COX-2 inhibitor, but this decrease was only associated with sclerotically degenerated arteries (39). These data confirm previous *in vivo* observations regarding COX-2 inhibitors, which may clearly influence the vascular system not only by limiting the synthesis of PGI_2_ and TXA_2_ (which appears the most distinct), but also by increasing the sensitivity to vasodilating as well as vasoconstricting factors. In conclusion, the present study identified high vasorelaxing efficacy of celecoxib at the background of the PDE inhibitors, which was observed not only in the presence, but also in the absence of the endothelium and may be evidence for relaxation caused by this COX-2 inhibitor in the cAMP- and cGMP-dependent pathways.

## Figures and Tables

**Table I:** Pharmacometric parameters of human mesenteric arteries (with and without endothelium) treated with PDE inhibitors or celecoxib and constricted by ET-1.

Treatments	No.	EC_50_ (M/L)	SE (EC_50_)	P-value (EC_50_)	pD_2_	Em (%)	SE (Em %)	P-value (Em)	RP1 (%)	RP2 (%)
Mesenteric arteries										
with endothelium										
Celecoxib	12	1.91E-07	0.08E-07	-	−6.72	67	5.6	-	1.7	0.45
Sildenafil	12	2.29E-09	0.04E-09	-	−8.64	44	6.3	-	138.4	1.35
Zaprinast	12	1.91E-06	0.05E-06	-	−5.72	58	4.9	-	0.2	7.24
Rolipram	12	3.22E-05	0.04E-05	-	−4.49	74	6.1	-	0.01	44.91
without endothelium										
Celecoxib	12	4.27E-05	0.04E-05	<0.001	−4.37	64	6.1	NS	0.01	0.45
Sildenafil	12	1.70E-07	0.07E-07	<0.001	−6.77	22	4.6	<0.001	1.9	1.35
Zaprinast	12	2.63E-05	0.04E-05	<0.001	−4.58	18	4.4	<0.001	0.01	7.24
Rolipram	12	7.17E-05	0.06E-05	<0.001	−4.14	69	5.8	0.052	0.00	44.91

Human mesenteric artery smooth muscle cells were treated with increasing concentrations of celecoxib, sildenafil, rolipram and zaprinast, for normal and endothelium-denudated arteries. PDE, phosphodiesterase; ET-1, endothelin-1; EC_50_, concentration causing an effect equal to half of the maximum effect; SE, standard error of the mean; pD_2_, negative common logarithm of EC_50_; Em, maximum effect; NS, no significance; RP, relative potency. RP1 with endothelium was calculated using the assumed control (reference) value of EC_50_ ET-1 and referred value, EC_50_ of an analyzed substance in the system with the endothelium. RP1 without endothelium was calculated as aforementioned, but in mesenteric arteries without endothelium. RP2 was calculated using the assumed control (reference) value of EC_50_ of an analyzed substance in the system with the endothelium and referred value, EC_50_ of an analyzed substance in the system without the endothelium.
